# Application of machine learning methods for the prediction of true fasting status in patients performing blood tests

**DOI:** 10.1038/s41598-022-15161-2

**Published:** 2022-07-13

**Authors:** Shih-Ni Chang, Ya-Luan Hsiao, Che-Chen Lin, Chuan-Hu Sun, Pei-Shan Chen, Min-Yen Wu, Sheng-Hsuan Chen, Hsiu-Yin Chiang, Chiung-Tzu Hsiao, Emily K. King, Chun-Min Chang, Chin-Chi Kuo

**Affiliations:** 1 Big Data Center, China Medical University Hospital and College of Medicine, China Medical University, 2, Yude Rd., North Dist., Taichung, 404 Taiwan; 2 The PhD Program for Cancer Biology and Drug Discovery, College of Medicine, China Medical University, Taichung, Taiwan; 3 Department of Health Policy and Management, Johns Hopkins University Bloomberg School of Public Health, Baltimore, MD USA; 4 Department of Laboratory Medicine, China Medical University Hospital, Taichung, Taiwan; 5 Department of Medical Media Design and Application, Interpedia Incorporated, Taichung, Taiwan; 6 Department of Electrical and Computer Engineering, University of Wisconsin-Madison, Madison, WI USA; 7 Division of Nephrology, Department of Internal Medicine, China Medical University Hospital and College of Medicine, China Medical University, Taichung, Taiwan

**Keywords:** Diabetes, Pre-diabetes, Epidemiology, Mathematics and computing, Computational science, Scientific data

## Abstract

The fasting blood glucose (FBG) values extracted from electronic medical records (EMR) are assumed valid in existing research, which may cause diagnostic bias due to misclassification of fasting status. We proposed a machine learning (ML) algorithm to predict the fasting status of blood samples. This cross-sectional study was conducted using the EMR of a medical center from 2003 to 2018 and a total of 2,196,833 ontological FBGs from the outpatient service were enrolled. The theoretical true fasting status are identified by comparing the values of ontological FBG with average glucose levels derived from concomitant tested HbA1c based on multi-criteria. In addition to multiple logistic regression, we extracted 67 features to predict the fasting status by eXtreme Gradient Boosting (XGBoost). The discrimination and calibration of the prediction models were also assessed. Real-world performance was gauged by the prevalence of ineffective glucose measurement (IGM). Of the 784,340 ontologically labeled fasting samples, 77.1% were considered theoretical FBGs. The median (IQR) glucose and HbA1c level of ontological and theoretical fasting samples in patients without diabetes mellitus (DM) were 94.0 (87.0, 102.0) mg/dL and 5.6 (5.4, 5.9)%, and 92.0 (86.0, 99.0) mg/dL and 5.6 (5.4, 5.9)%, respectively. The XGBoost showed comparable calibration and AUROC of 0.887 than that of 0.868 in multiple logistic regression in the parsimonious approach and identified important predictors of glucose level, home-to-hospital distance, age, and concomitantly serum creatinine and lipid testing. The prevalence of IGM dropped from 27.8% based on ontological FBGs to 0.48% by using algorithm-verified FBGs. The proposed ML algorithm or multiple logistic regression model aids in verification of the fasting status.

## Introduction

With the universal implementation of electronic medical records (EMRs), researchers have actively leveraged real-world EMR data in diabetes research and management in clinical practice^1^. The development of algorithms to identify patients with prediabetes and diabetes mellitus (DM) with high validity has become increasingly fundamental in improving the patients’ quality of care and preventing complications associated with DM. In current clinical practice, the phenotypes of DM are defined by various combinations of the different components of the EMR, such as diagnostic codes, medication data, and laboratory values related to glucose homeostasis^2^. Thus, current diagnostic algorithms have yielded significant variation in the validity for identification of DM^2^. In recent years, several studies have indicated that machine learning (ML) algorithms may better identify diabetic status in EMRs for cohort establishment^3,4^. Other studies applied ML techniques to predict DM or undiagnosed DM based on clinical information^5–8^. Systematic reviews reported that most ML studies used the supervised learning approach and a comparison of the approaches indicated that support vector machine (SVM) was the most widely used algorithm^9,10^. Deep learning (DL) models such as artificial neural networks (ANNs) and deep neural networks (DNNs) have been applied and reported in some studies showing superior performance than conventional ML approaches in predicting DM-related phenotypes^11,12^. However, these studies usually assumed that fasting blood glucose (FBG) values are valid if labeled as such by the clinical laboratory, which may lead to potential overestimation of fasting status^13^. As demonstrated in a survey conducted by Tseng et al., only approximately half of the patients reported to have adequately fasted before phlebotomy at a large academically affiliated hospital^13^. Another study surveyed around 150 outpatients and stated that 40% did not fast before going to the hospital for laboratory blood work^14^. Both studies pointed out that documentation of fasting state before phlebotomy was often non-existent as these data are not routinely collected by healthcare providers or the laboratory team and recorded in the EMR. Similarly, information regarding whether patients had been given instructions to fast before phlebotomy was also not recorded^13,14^. Despite the importance of the fasting status in patients undergoing phlebotomy, there has been relatively few research conducted in the current literature to verify the fasting status of patients before blood work. The lack of knowledge of the fasting state of patients presents a challenge for healthcare providers in determining whether patients had truly fasted before laboratory blood testing and may prohibit them from interpreting the results in accordance with diabetes screening guidelines, resulting in missed diagnoses of prediabetes and type 2 diabetes.

Misclassification of fasting status negatively influences the clinical accuracy of conventional or ML models in screening DM or predicting the risk of DM^15^. Verification of fasting blood samples is therefore a significant challenge in analyzing real-world EMR data for epidemiological research, particularly when the disease diagnostic criteria are based on fasting blood samples. The current reference standard for confirmation of the fasting status relies on self-reported information from the patients during phlebotomy, which may be influenced by recall and awareness biases. To the best of our knowledge, no studies have used EMR data to investigate the discordance between prescribed and actual fasting status based on the distribution of BG and concomitant HbA1c values. Using a large clinical data repository of more than 2.75 million patient records from a tertiary medical center in central Taiwan, we systematically evaluated the distribution of BG values. We used the HbA1c-estimated average glucose level to define fasting status, followed by the development of prediction models using ML.

## Materials and methods

### Study data source and sample selection

**Figure 1:**
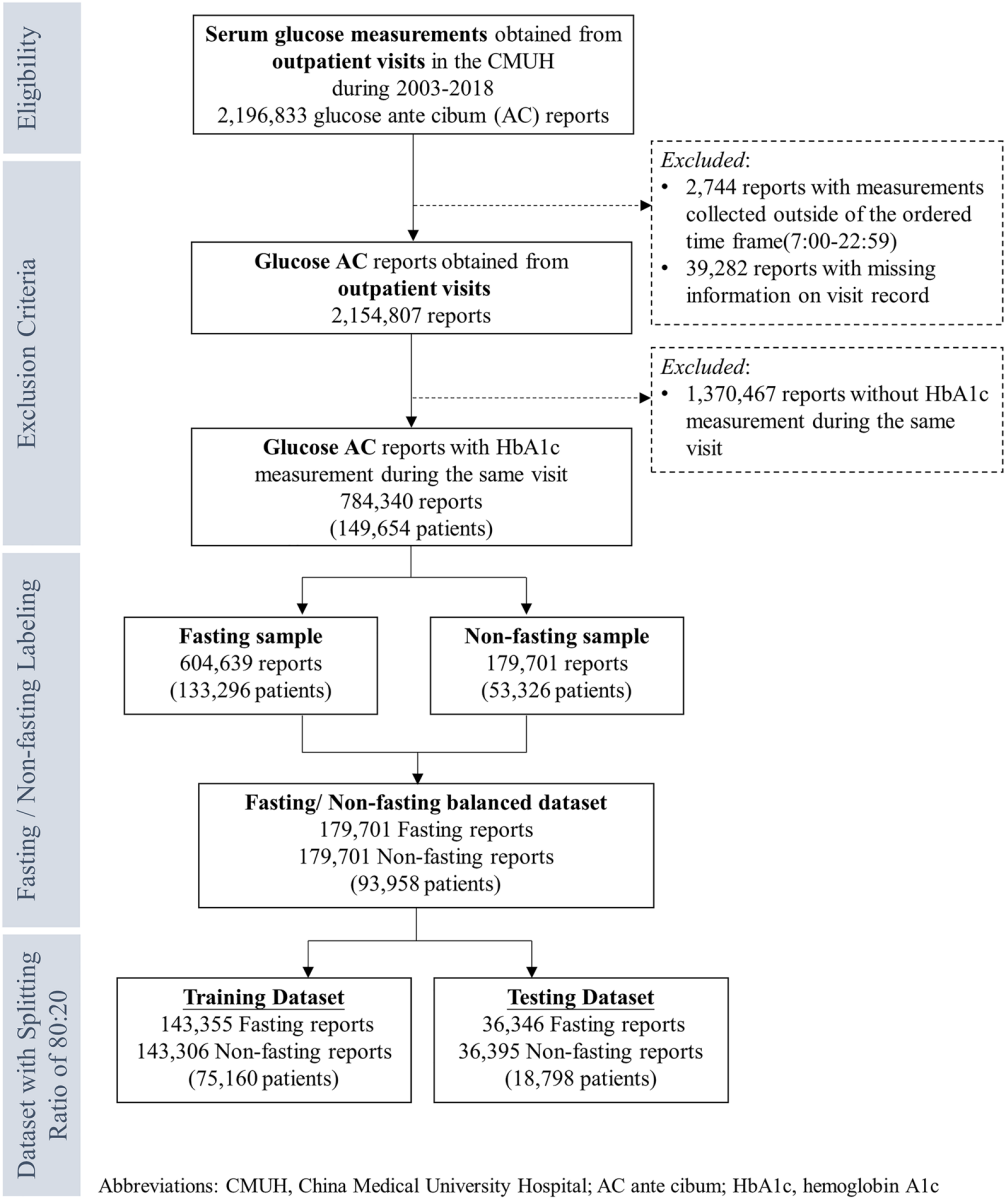
Sample selection process from ontological glucose ante cibum (AC) to theoretical fasting classification, followed by splitting of the dataset into training and testing datasets.

The China Medical University Hospital (CMUH) Clinical Research Data Repository (CRDR) carefully validated the EMRs of 2,873,887 patients who had sought care at CMUH between January 1, 2003, and December 31, 2018. The methodologic details have been published elsewhere^16–19^. Of the 2,873,887 patients, 945,792 underwent glucose measurements using sera samples from inpatient and outpatient services. The sample selection flow is summarized in Fig. 1. All methods in this study were performed in accordance with the relevant guidelines/regulations. This study protocol was approved by the Big Data Center of China Medical University Hospital and the Research Ethical Committee/Institutional Review Board of China Medical University Hospital (CMUH105-REC3-068) and the need to obtain informed consent for the present study was waived by the Research Ethical Committee of China Medical University Hospital.

### Sociodemographic and clinical variables

The covariables of interest were obtained from the CRDR, including patient demographics, specifically age and sex, and body mass index, which was calculated as the weight in kilograms divided by the height in square meters. The presence of hypertension or type 2 DM was captured based on associated ICD-9/-10 codes or the use of glucose-lowering medications or antihypertensive agents. A history of cardiovascular disease was also documented if the patients had a record of coronary artery disease, myocardial infarction, stroke or congestive heart failure in EMRs based on International Classification of Diseases (ICD) 9th and 10th edition codes. All other coexisting comorbidities were also captured based on ICD-9/-10 codes from the repository or EMR data. Additional provider- or patient-level factors such as medication records, health care provider specialty, and biochemical measures were obtained from repository data or the EMRs within a 1-year window prior to enrollment into the study cohort.

Another patient-level factor that we included was the distance from the patients’ home to the hospital as we hypothesized that fasting status might be associated with the travel time to the healthcare facility. Currently, no studies have investigated the association of distance between healthcare facilities and homes and fasting status. However, a few studies have provided evidence that increasing travel distance to the primary care provider may affect and decrease glycemic control^20–22^.Therefore, we calculated the straight-line distance between hospital to home as it is the most common method for this type of calculation^23^. The home-to-hospital distance was calculated in two steps. First, a geocoding application programming interface developed by Google Maps was used to transform the map coordinates of the entire study population’s home addresses and locations. The distance between the homes and the hospital was calculated using the geographic information system (ArcGIS version 10; ESRI, Redlands, CA, USA).

### Determination of glucose and HbA1c levels

Blood glucose levels were determined by the central laboratory using the Beckman Oxygen electrode (glucose oxidase method) with a Beckman Synchron^®^ LX20 and Beckman UniCel^®^ DxC 800 (Beckman Coulter Inc., Brea, CA, USA) from January 1, 2003, to September 30, 2007, and from October 1, 2007, to December 31, 2018, respectively. The level of HbA1c was measured using boronate affinity and high-performance liquid chromatography (HPLC) methods with the Primus CLC385 analyzer from January 1, 2003, to June 30, 2008, cation exchange HPLC methods with the Tosoh HLC-723 G7 (Tosoh Corporation, Tokyo, Japan) from July 31, 2008, to December 31, 2013, and boronate affinity and HPLC methods with the Trinity Biotech Premier Hb9210 from January 1, 2014, to December 31, 2018.

From the CMUH-CRDR laboratory database, we selected the glucose measurements specified as fasting glucose (AC, ante cibum), postprandial glucose (PC, post cibum), and random glucose. We excluded data recorded as nonnumerical values, values higher than 1000 mg/dL, or zero values. All glucose measurements could also be classified as inpatient, outpatient clinic, and emergency department services. Only measurements obtained in the outpatient setting were included in the final analysis. The HbA1c-derived averaged glucose level (AC_average_) was defined based on Nathan et al.’s formula as a theoretical upper limit of fasting glucose^24^.

### Data conditioning steps to determine ontological fasting glucose

To investigate the “true” ontological fasting status on blood glucose measurements, we filtered glucose measurements that were highly likely nonfasting in the outpatient setting to derive ontological fasting glucose (AC_ontological_) as follows. Glucose measurements were reclassified as non-AC_ontological_ if:

1. the data were labeled as post cibum glucose or random glucose,
2. the glucose measurement included additional descriptions/labels such as “one-touch”, “bedside check”, or “PC” or contained descriptions indicating active food intake before phlebotomy, regardless of the laboratory test prescribed (e.g., fasting glucose),
3. patients had multiple fasting glucose measurements on the same day; only the first measurement was considered as non-AC_ontological_.

### Definition of theoretical fasting status

Three criteria were used to define the theoretical fasting status (AC_theoretical_) of patients who underwent concomitant AC_ontological_ and HbA1c measurements on the same day: (1) an AC_ontological_ < 100 md/dL in patients without DM with HbA1c < 5.5%; (2) an AC_ontological_ < AC_average_ − 1 standard deviation of AC_ontological_ glucose in patients without DM with an HbA1c between 5.5 and 6.4%; and (3) an AC_ontological_ < AC_average_ in patients with DM. Once the patients' glucose AC was defined as AC_theoretical_, the corresponding blood samples were defined as fasting samples. Otherwise, they were considered nonfasting samples. These criteria are based on the physiological profiling of glucose and insulin variation over 24 h in individuals with and without diabetes^25,26^. The A1c-derived estimated average glucose (AC_average_) summarizes the daily glucose variation over the past 90 days, depicting an averaged value between the lowest and the highest glucose level in this time window among patients with a stable metabolic state. Therefore, if truly obtained in the fasting status, the glucose level should be theoretically less than the level of AC_average_^27^. To verify the validity of our proposed criteria, we used the glucose AC from 4519 patients who provided morning fasting samples before the procedure of pan-endoscopy in CRDR as the true fasting glucose AC and only 314 measurements (6.95%) were misclassified as nonfasting based on our criteria.

### Statistical analysis

The clinical characteristics of patients with a theoretical fasting sample and those with a theoretical nonfasting sample were compared. The probability densities of glucose levels between fasting and nonfasting status were examined based on the diabetic status. We also assessed whether the levels of fasting glucose differed if the glucose measurements were taken at the same time with lipid profiles. Conventional logistic regression and ML were applied to develop a tool for predicting whether the glucose measurements were fasting measures. We tested model discrimination and calibration using area under the receiver operating characteristic (AUROC) statistics and calibration curves.

### Machine learning approach and evaluation

To use ML for predicting whether the blood samples were obtained in the fasting state, a balanced dataset was curated to obtain a 1:1 ratio of AC_ontological_ and AC_theoretical_, which was composed of 93,958 patients (Fig. 1). Patients within this balanced dataset were separated into training and testing sets at an 80/20 proportion while maintaining a 1:1 ratio of AC_ontological_ and AC_theoretical_. The demographic, clinical, and biochemical information of the patients, such as age, ICD-9 or -10 codes, medication histories, and laboratory test results, was then extracted from the CMUH-CRDR. We applied logistic regression and eXtreme Gradient Boosting model (XGBoost), a scalable end-to-end tree boosting model proposed by Chen and Guestrin^28^, to evaluate the performance of predicting fasting status. We additionally experimented with two efficient algorithms, CatBoost and ensemble models with H2O AutoML, to better handle the categorical variables and explore the predictive performance using multiple learning algorithms^29,30^. The objective function of this binary classification problem was to minimize binary entropy loss; the hyperparameters of our XGBoost model were determined using the Tree of Parzen Estimators (TPE) method^31^. Taking the implementation of XGBoost in Python as an example, the finalized hyperparameters were set as tree depth = 8, learning rate = 0.1, gamma = 0.5, minimum sum of instance weight = 7, number of estimators = 300, and the remaining parameters were set using the default setting. Detailed parameter ranges for grid search were summarized in Supplementary Table 1. To implement ensemble models with H2O AutoML in Python, we stacked various algorithms, such as XGBoost, Random Forest, and Gradient Boosting Machines. The model output of XGBoost, CatBoost, or Ensemble models was the probability of AC_theoretical_. The performance quantification of each ML algorithm was evaluated in terms of AUROC, accuracy, precision, recall, and F1 score using a fivefold cross-validation scheme. We used the bootstrapping method with 2000 repetitions to statistically test the difference between the paired AUROCs^32^. Finally, we compared the proportion of glucose AC ≥ 126 mg/dL calculated with or without the ML algorithm to classify the fasting status. We also classified glucose AC ≥ 126 mg/dL, regardless of ontological or predicted fasting samples, which did not lead to the diagnosis of diabetes over the study periods as ineffective glucose measurements (IGM). All statistical analyses were performed using SAS version 9.4 (SAS Institute Inc., Cary, NC, USA), R version 3.5.1 (R Foundation for Statistical Computing, Vienna, Austria), and Python version 3.7.3 under a Linux operating system. The Python package version was 1.5.2 for XGBoost, 1.0.4 for CatBoost, and 3.36.0.2 for H2O AutoML. The two-sided statistical significance level of α was set at 0.05.

### Ethical approval

The study was approved by the Research Ethical Committee/Institutional Review Board of China Medical University Hospital (CMUH105-REC3-068).

## Results

### Distribution of glucose level by fasting and diabetic status

A total of 359,402 AC_ontological_ data points were included in the final analysis, with a mean sample age of 59.7 ± 14.6 years. Approximately half of the sample population were female (46.1%). When restricting to only the patients’ first sample in the CRDR (n = 93,958), the average age was 54.4 ± 15.5 years, and 45.9% of these patients were female. Of these 93,958 patients, 29.2% had been diagnosed with DM at the first AC_ontological_. Blood glucose measurements considered to be collected during fasting state were observed in younger non-DM patients but not among younger patients with DM. Nonfasting samples were more likely to be provided by male patients, regardless of their diabetic status. Moreover, samples were more likely to be fasting measures if the lipid profiles of the patients were concomitantly examined. Statistical differences were observed for the majority of the biochemical measures between fasting and nonfasting samples. Specifically, levels of triglyceride demonstrated clinically significantly different results (> 15 mg/dL) between fasting and nonfasting samples, regardless of the diabetic status (Table 1).

**Table 1:** Comparison of demographic and biochemical profiles of ontologically fasting samples with concomitant HbA1c measurement according to DM and theoretical fasting status.

Variables	Non-DM				DM			
	Overall	Fasting	Nonfasting	*p*	Overall	Fasting	Nonfasting	*p*
	n = 118,383	n = 53,080	n = 65,303		n = 241,019	n = 126,621	n = 114,398	
Age, years	55.3 (15.0)	50.7 (15.6)	59.1 (13.4)	< 0.01	61.9 (13.9)	62.6 (13.3)	61.1 (14.6)	< 0.01
Male	66,333 (56.0)	29,147 (54.9)	37,186 (56.9)	< 0.01	127,301 (52.8)	66,791 (52.8)	60,510 (52.9)	0.47
BMI, kg/m^2^	25.4 (4.85)	24.8 (4.83)	26.1 (4.78)	< 0.01	26.1 (4.63)	26.2 (4.65)	25.9 (4.60)	< 0.01
**Sampling timing of the day**				< 0.01				< 0.01
7:00–12:59	109,784 (92.7)	48,168 (90.8)	61,616 (94.4)		225,770 (96.7)	119,210 (94.2)	106,560 (93.2)	
13:00–17:59	7465 (6.31)	4322 (8.14)	3143 (4.81)		11,761 (4.88)	5805 (4.58)	5956 (5.21)	
18:00–22:59	1134 (0.96)	590 (1.11)	544 (0.83)		3488 (1.45)	1606 (1.27)	1882 (1.65)	
Interval between request and sampling day, day	32.0 (40.8)	24.9 (37.9)	37.8 (42.2)	< 0.01	57.6 (34.2)	58.71 (33.3)	56.39 (35.2)	< 0.01
No. of outpatient visits	9.42 (17.6)	7.44 (15.8)	11.0 (18.7)	< 0.01	19.56 (24.7)	19.07 (23.7)	20.11 (25.8)	< 0.01
Distance to hospital, km	15.4 (76.3)	17.7 (89.7)	13.6 (63.9)	< 0.01	10.6 (55.4)	10.6 (51.7)	10.59 (59.2)	0.87
Concomitant lipid testing	95,750 (80.9)	47,735 (89.9)	48,015 (73.5)	< 0.01	147,021 (61.0)	81,320 (64.2)	65,701 (57.4)	< 0.01
**Division**				< 0.01				< 0.01
General medicine	19,268 (16.3)	6913 (13.02)	12,355 (18.92)		19,988 (8.29)	10,277 (8.12)	9711 (8.49)	
Metabolism/endocrinology	14,190 (12.0)	3553 (6.69)	10,637 (16.29)		132,971 (55.2)	70,496 (55.67)	62,475 (54.61)	
Nephrology	7461 (6.30)	2070 (3.9)	5391 (8.26)		21,015 (8.72)	10,298 (8.13)	10,717 (9.37)	
Cardiology	20,837 (17.6)	6260 (11.79)	14,577 (22.32)		36,060 (15.0)	20,265 (16)	15,795 (13.81)	
Family medicine	10,810 (9.13)	4708 (8.87)	6102 (9.34)		16,022 (6.65)	8243 (6.51)	7779 (6.8)	
Health management center	34,572 (29.2)	25,217 (47.51)	9355 (14.33)		766 (0.32)	458 (0.36)	308 (0.27)	
Surgery	5469 (4.62)	2782 (5.24)	2687 (4.11)		2915 (1.21)	1552 (1.23)	1363 (1.19)	
Pediatrics	1109 (0.94)	710 (1.34)	399 (0.61)		3607 (1.50)	1065 (0.84)	2542 (2.22)	
Chinese medicine	4232 (3.57)	735 (1.38)	3497 (5.36)		7226 (3.00)	3753 (2.96)	3473 (3.04)	
Other	435 (0.37)	132 (0.25)	303 (0.46)		449 (0.19)	214 (0.17)	235 (0.21)	
**Comorbidity**								
Hypertension	33,868 (28.6)	10,916 (20.6)	22,952 (35.2)	< 0.01	143,831 (59.7)	76,406 (60.34)	67,425 (58.94)	< 0.01
Coronary artery disease	12,906 (10.9)	3981 (7.50)	8925 (13.7)	< 0.01	46,568 (19.3)	24,982 (19.73)	21,586 (18.87)	< 0.01
Stroke	8713 (7.36)	3236 (6.10)	5477 (8.39)	< 0.01	31,375 (13.0)	16,602 (13.11)	14,773 (12.91)	0.15
**Biochemical variables**								
Glucose, mg/dL	124 (47.6)	96.9 (19.5)	145 (52.3)	< 0.01	163 (62.5)	132 (36.7)	197 (67.1)	< 0.01
HbA1c, %	6.48 (1.44)	5.86 (0.98)	6.97 (1.57)	< 0.01	7.55 (1.50)	7.59 (1.42)	7.50 (1.58)	< 0.01
Hemoglobin, g/dL	14.0 (1.97)	14.1 (1.77)	13.7 (2.23)	< 0.01	11.8 (2.29)	12.0 (2.21)	11.6 (2.35)	< 0.01
Total cholesterol, mg/dL	191 (41.5)	193 (39.4)	190 (43.6)	< 0.01	177 (43.5)	174 (41.6)	180 (45.5)	< 0.01
LDL, mg/dL	114 (34.6)	116 (33.7)	111 (35.4)	< 0.01	97.2 (33.0)	96.6 (32.2)	97.9 (33.9)	< 0.01
HDL, mg/dL	47.8 (13.7)	49.9 (14.1)	45.2 (12.6)	< 0.01	43.6 (12.5)	43.6 (12.2)	43.6 (12.8)	0.95
Triglyceride, mg/dL	148 (168)	125 (114)	172 (208)	< 0.01	179 (231)	160 (191)	200 (269)	< 0.01
BUN, mg/dL	14.6 (12.7)	12.3 (8.83)	18.2 (16.3)	< 0.01	33.0 (24.3)	31.0 (23.2)	35.0 (25.3)	< 0.01
Serum creatinine, mg/dL	1.14 (1.51)	1.00 (1.09)	1.28 (1.82)	< 0.01	1.63 (2.20)	1.53 (1.99)	1.76 (2.41)	< 0.01
Serum sodium, mmol/L	139 (3.61)	140 (3.22)	138 (3.86)	< 0.01	137 (3.83)	138 (3.41)	136 (4.06)	< 0.01
Serum potassium, mmol/L	4.09 (0.52)	4.02 (0.49)	4.15 (0.54)	< 0.01	4.33 (0.61)	4.34 (0.58)	4.32 (0.64)	< 0.01
AST, IU/L	30.5 (26.0)	27.4 (19.2)	34.9 (32.8)	< 0.01	33.1 (29.1)	32.1 (25.6)	34.1 (32.1)	< 0.01
ALT, IU/L	33.1 (33.1)	30.0 (29.6)	36.4 (36.0)	< 0.01	31.1 (28.7)	30.4 (26.6)	31.9 (30.8)	< 0.01
Uric acid, mg/dL	6.00 (1.59)	5.89 (1.55)	6.13 (1.63)	< 0.01	6.29 (1.80)	6.26 (1.78)	6.32 (1.83)	< 0.01
Albumin, g/dL	4.50 (0.41)	4.55 (0.35)	4.41 (0.48)	< 0.01	3.97 (0.54)	4.03 (0.50)	3.92 (0.56)	< 0.01
Estimated blood osmolality	291 (7.90)	290 (7.02)	293 (8.68)	< 0.01	296 (10.1)	294 (9.48)	297 (10.5)	< 0.01
Urine specific gravity	1.02 (0.01)	1.02 (0.01)	1.02 (0.01)	0.13	1.02 (0.01)	1.02 (0.01)	1.02 (0.01)	< 0.01
Urine pH	6.01 (0.68)	6.03 (0.70)	5.98 (0.66)	< 0.01	6.01 (0.63)	6.03 (0.65)	5.99 (0.62)	< 0.01

Values for continuous and categorical variables are expressed as mean (standard deviation) and frequency (%), respectively. Levels of glucose, HbA1c, and other biochemical variables were measured on the same day. P value indicates the significant difference of variables between theoretically fasting and nonfasting samples. *BMI* body mass index, *LDL* low-density lipoprotein, *HDL* high-density lipoprotein, *BUN* blood urea nitrogen, *AST* aspartate aminotransferase, *ALT* alanine transaminase.

**Figure 2:**
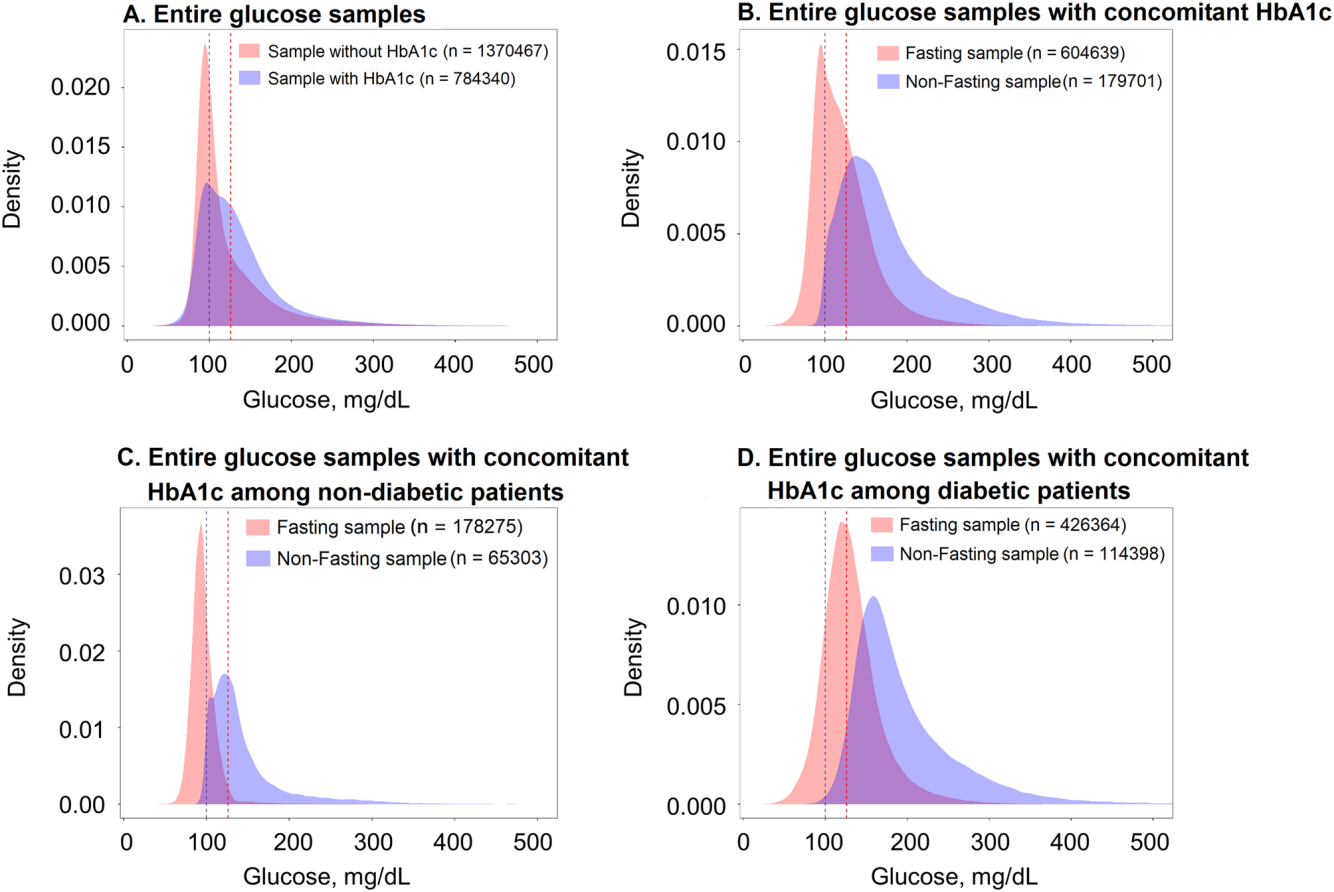
Density plots of ontological glucose AC in selected samples as follows: (**A**) entire samples stratified by the availability of HbA1c measured on the same day; (**B**) samples with HbA1c measured on the same day, stratified by theoretical fasting and nonfasting status; (**C**) the entire samples with A1c measured on the same day, stratified by fasting and nonfasting status in patients without DM; (**D**) the entire samples with HbA1c measured on the same day, stratified by fasting and nonfasting status in patients with DM. The dark blue dashed line shows the glucose value at 100 mg/dL, and the red dashed line shows the glucose value at 126 mg/dL.

**Figure 3:**
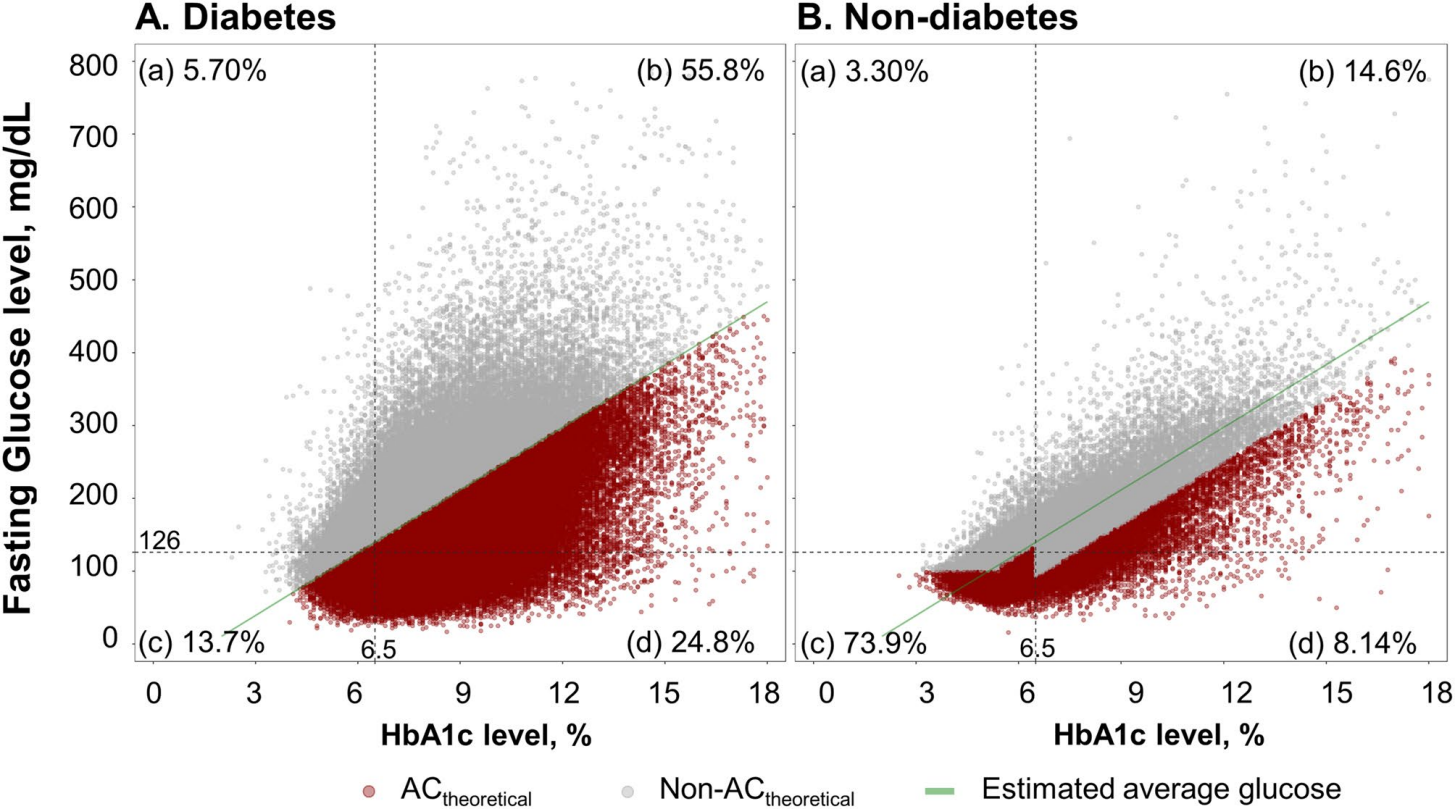
Scatter plot of HbA1c and fasting glucose levels. The figure is divided into four quadrants (a, b, c, and d) according to the diagnostic criteria of the American Diabetes Association (ADA) by diabetic status.

The peak of the density curves of AC_ontological_ with and without same-day HbA1c measures was similar at approximately 100 mg/dL. However, the width of the distribution of AC_ontological_ with HbA1c measures was wider than those without the HbA1c measures (Fig. 2A). Peaks of the density curves of AC_theoretical_ and nonfasting AC_ontological_ were separated with a peak value slightly lower than 100 and slightly above 126 mg/dL, respectively (Fig. 2B). Among patients without DM, a peak shift to the left to < 126 mg/dL was noted in the nonfasting samples compared with the entire sample with concomitant HbA1c (Fig. 2C). By contrast, among patients with DM, the peak of the fasting samples shifted right to approximately 126 mg/dL (right-shifting; Fig. 2D). Figure 3 shows the scatter plots of AC_ontological_ and HbA1c based on diabetic status and highlights the distribution of AC_theoretical_.

### Factors associated with nonfasting status

The entire dataset consisted of 67 attributes (Supplementary Table 2) and details of relevant missingness are provided in Supplementary Table 3. In multiple logistic regression, age, male sex, distance from the home to the hospital, the timing of blood sampling, and the cumulative frequency of outpatient visits 1 year prior to the blood sampling were associated with a higher probability of being in a nonfasted state. Patients with a history of DM, hypertension or coronary artery disease, statin medication, and concomitant lipid and glucose testing were significantly associated with the fasting status. Comparing the odds of nonfasting status among patients who visited the Health Management Center, those who were ordered glucose measurement in the departments of metabolism and endocrinology, general medicine, and nephrology were twice as likely to be in a nonfasted state (Table 2). In addition, patients who underwent concomitant glucose and lipid testing were more likely to follow the fasting instruction, with the odds ratio of being in a nonfasted state of 0.78 (95% CI 0.76–0.80).

**Table 2:** Odds ratios (95% confidence intervals) of being in the theoretically nonfasting status using the AC_ontological_ sample in the training dataset (n = 277,822*).

Variables	Univariate analysis		Model 1		Model 2	
	OR (95% CI)	*p*-value	OR (95% CI)	*p*-value	OR (95% CI)	*p*-value
Glucose, per 5 mg/dL	1.20 (1.20–1.21)	< 0.001	1.16 (1.16–1.16)	< 0.001	1.23 (1.23–1.23)	< 0.001
Age, per 5 years	1.16 (1.16–1.17)	< 0.001	1.01 (1.00–1.01)	< 0.001	1.05 (1.04–1.05)	< 0.001
Male	1.05 (1.03–1.06)	< 0.001	1.09 (1.07–1.11)	< 0.001	1.16 (1.14–1.18)	< 0.001
**Timing of the day**						
7:00–12:59	Ref		Ref		Ref	
13:00–17:59	0.96 (0.93–1.00)	0.03	0.90 (0.86–0.94)	< 0.001	0.87 (0.83–0.91)	< 0.001
18:00–22:59	1.14 (1.07–1.22)	< 0.001	0.76 (0.70–0.84)	< 0.001	0.78 (0.70–0.86)	< 0.001
Interval between request and sampling, per 28 day	1.07 (1.05–1.08)	< 0.001	0.95 (0.94–0.96)	< 0.001	1.08 (1.07–1.08)	< 0.001
No. of outpatient visits, per 4 visits	1.03 (1.02–1.04)	< 0.001	0.99 (0.99–1.00)	< 0.001	1.02 (1.02–1.02)	< 0.001
Distance from home to hospital, per 10 km	0.998 (0.996–1.000)	0.04	0.998 (0.997–1.000)	0.03	0.998 (0.996–1.00)	0.01
**Division**						
Health management center	Ref		Ref		Ref	–
General medicine	3.39 (3.27–3.52)	< 0.001	1.49 (1.43–1.56)	< 0.001	2.24 (2.14–2.35)	< 0.001
Metabolism/endocrinology	2.57 (2.49–2.65)	< 0.001	0.67 (0.65–0.70)	< 0.001	2.11 (2.02–2.21)	< 0.001
Nephrology	3.45 (3.32–3.58)	< 0.001	1.19 (1.13–1.25)	< 0.001	2.38 (2.25–2.51)	< 0.001
Cardiology	3.03 (2.93–3.13)	< 0.001	1.09 (1.04–1.14)	< 0.001	1.99 (1.89–2.08)	< 0.001
Family medicine	2.80 (2.69–2.91)	< 0.001	1.03 (0.98–1.08)	0.22	1.90 (1.81–2.00)	< 0.001
Surgery	2.57 (2.43–2.72)	< 0.001	1.30 (1.22–1.39)	< 0.001	1.74 (1.62–1.87)	< 0.001
Pediatrics	4.43 (4.13–4.77)	< 0.001	0.67 (0.60–0.75)	< 0.001	1.78 (1.59–1.99)	< 0.001
Chinese medicine	4.07 (3.87–4.28)	< 0.001	1.00 (0.94–1.06)	0.92	1.86 (1.74–1.99)	< 0.001
Other	3.98 (3.41–4.64)	< 0.001	1.30 (1.09–1.56)	0.004	1.95 (1.60–2.37)	< 0.001
Hypertension	1.04 (1.03–1.06)	< 0.001			1.02 (1.00–1.05)	0.03
Diabetes mellitus	0.70 (0.68–0.71)	< 0.001			0.09 (0.09–0.09)	< 0.001
Coronary artery disease	1.07 (1.05–1.09)	< 0.001			0.94 (0.91–0.96)	< 0.001
Stroke	1.02 (0.99–1.04)	0.12			0.99 (0.96–1.02)	0.64
Statin use	0.78 (0.77–0.80)	< 0.001			0.82 (0.80–0.83)	< 0.001
Concomitant lipid profile test	0.69 (0.68–0.70)	< 0.001			0.78 (0.76–0.80)	< 0.001
AIC			297,987		262,835	
AUC			0.820 (0.819–0.822)		0.867 (0.866–0.869)	
*p*-value for AUC difference			Ref		< 0.001	

*Sample were reduced because of missingness for the variable of "Distance from home to hospital".

*AIC* Akaike information criterion, *AUC* area under the curve.

### Machine learning performance in fasting status identification

**Figure 4:**
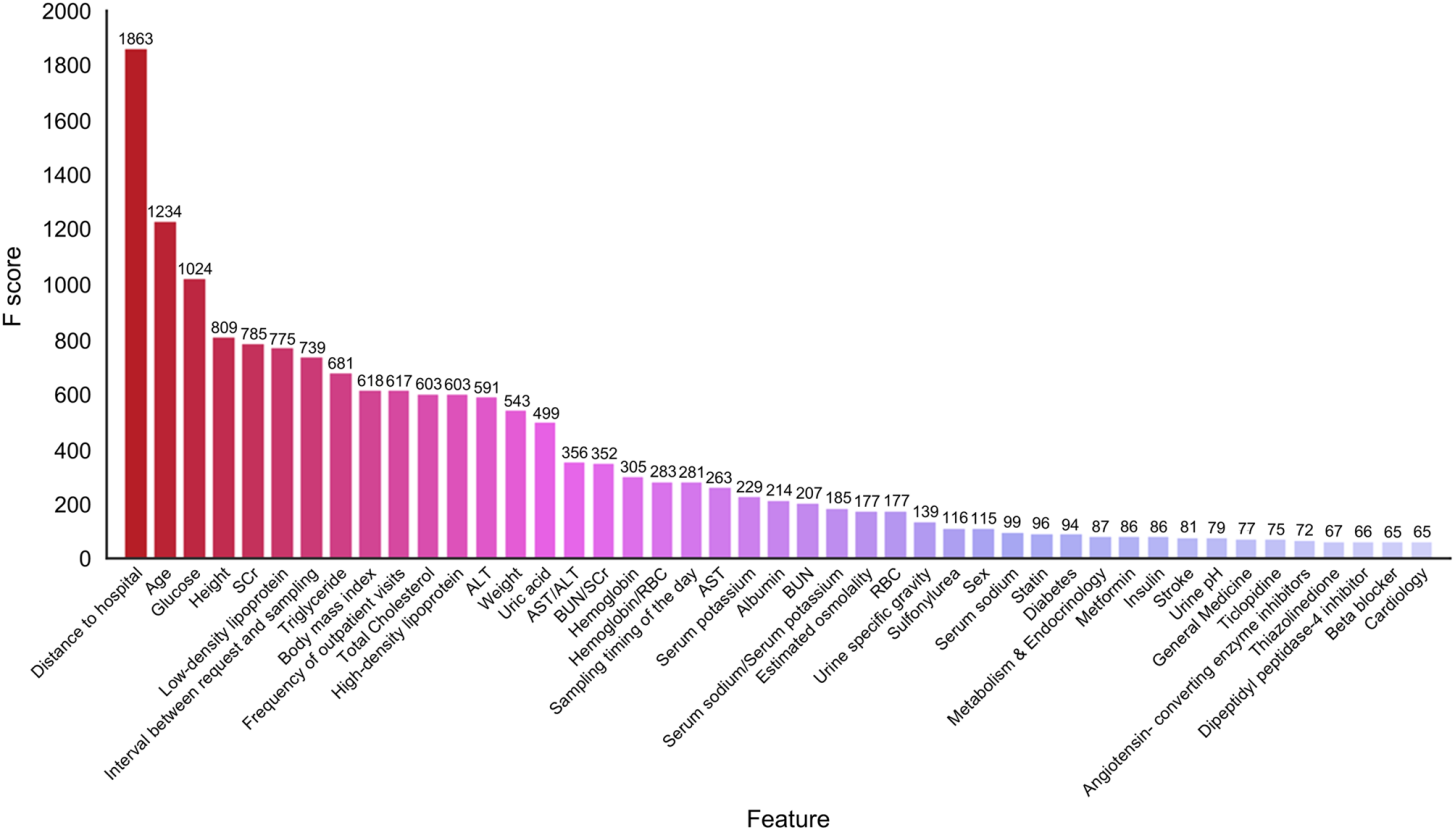
Top-ranked 45 features identified using the proposed XGBoost algorithm. *SCr* serum creatinine, *ALT* alanine transaminase, *AST* aspartate aminotransferase, *BUN* blood urea nitrogen, *RBC* red blood cell counts.

**Figure 5:**
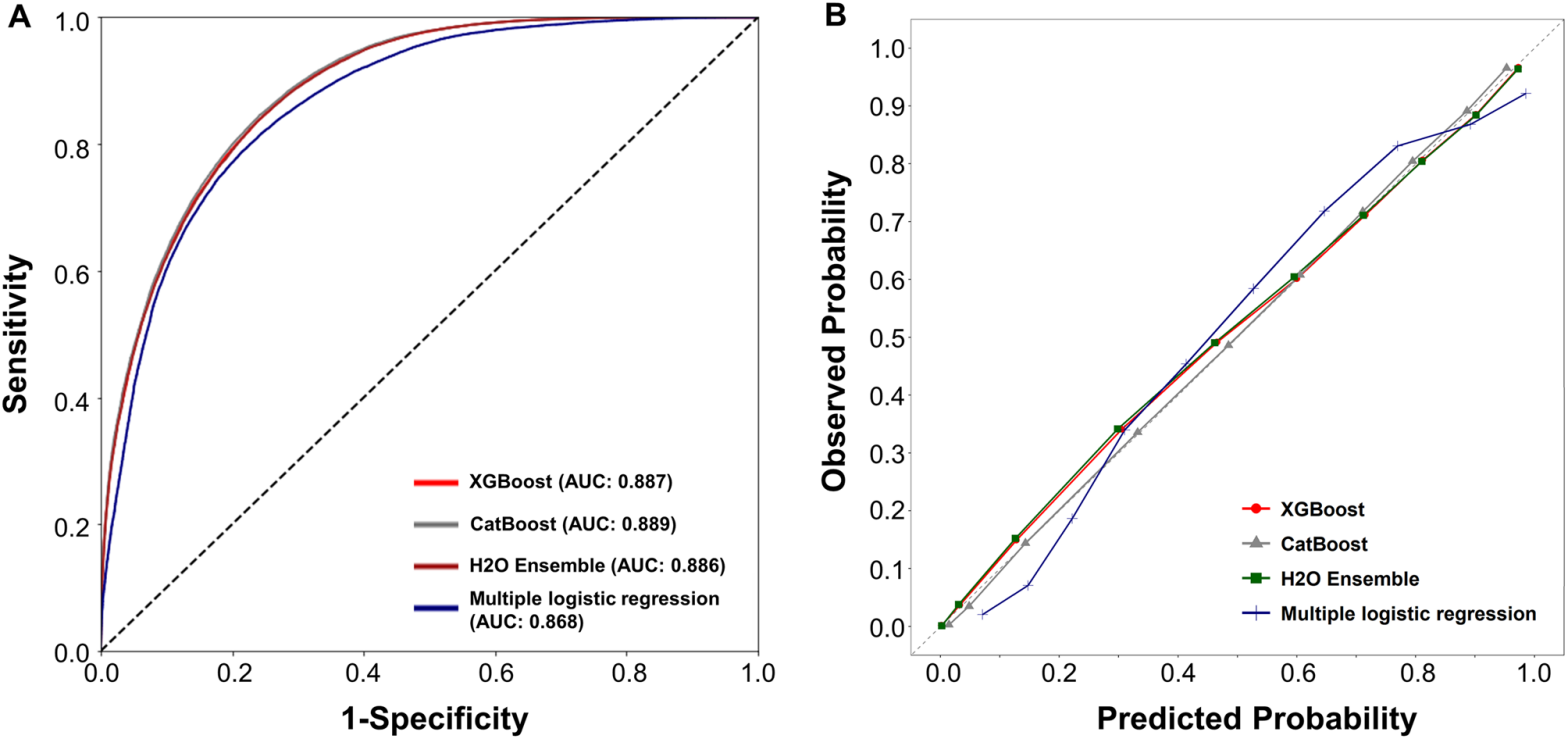
Discrimination statistics (**A**) and calibration plots (**B**) for multivariable logistic regression model and the parsimonious machine learning models in the testing dataset.

We conducted experiments on feature selection by building XGBoost models with the top 10, 25, 35, 45 features and found that using all 67 features generated the most accurate result. Compared with the predictive performance of multiple logistic regression for nonfasting status in the testing dataset, XGBoost with full features showed better sensitivity (77.8% vs. 76.1%), accuracy (80.9% vs. 78.5%), and F1 score (81.6% vs. 78.0%; Table 3). The top 45 scoring variables are summarized in Fig. 4. The level of the AC_ontological_, the distance from home to the hospital, age, height, and the level of serum creatinine were the most important features. When we used 14 features of the parsimonious model (model 2 in Table 2) in the XGBoost algorithm, the predictive performance was statistically better than that of the predictive model derived from multiple logistic regression. By contrast, the precision of the conventional logistic regression model was marginally better than the ML-based models (Table 3). The AUROC and calibration performance of our proposed ML methods were generally better than those of the multiple logistic regression model (AUROC 0.887 vs. 0.868, *p* < 0.001; Fig. 5). In the sensitivity analysis of other ML algorithms, the predictive performance was consistent with the original XGBoost (Table 3). However, the overall predictive performance difference between ML-based and conventional logistic regression models was not clinically relevant. The performance of different ML methods in the training dataset is provided in Supplementary Table 4.

**Table 3:** Comparison of performance of determining fasting status by XGBoost, CatBoost, H_2_O Ensemble and logistic regression models in the testing dataset (n = 70,644).

Algorithm/modeling strategy	Feature	Sensitivity	Specificity	Precision	F1-score	Accuracy	AUC
**Parsimonious modeling**							
Logistic regression	Model 2*	0.7608	0.8084	0.8081	0.7804	0.7845	0.868 (0.865–0.870)
XGBoost	Model 2*	0.8261	0.7700	0.7844	0.8047	0.7982	0.887 (0.885–0.890)
CatBoost	Model 2*	0.8415	0.7614	0.7813	0.8103	0.8017	0.889 (0.887–0.892)
H2O Ensemble	Model 2*	0.8823	0.7093	0.7546	0.8135	0.7964	0.886 (0.884–0.889)
**Full modeling**							
XGBoost	67	0.8394	0.7785	0.7934	0.8158	0.8092	0.896 (0.894–0.898)
CatBoost	67	0.8511	0.7574	0.7805	0.8142	0.8046	0.892 (0.890–0.894)
H2O Ensemble	67	0.8770	0.7399	0.7735	0.8220	0.8089	0.897 (0.894–0.899)
**Feature selection modeling**							
XGBoost	Top 45	0.8369	0.7789	0.7932	0.8145	0.8081	0.895 (0.892–0.897)
XGBoost	Top 35	0.8413	0.7735	0.7901	0.8149	0.8076	0.894 (0.892–0.897)
XGBoost	Top 25	0.8414	0.7706	0.7880	0.8138	0.8062	0.893 (0.891–0.896)
XGBoost	Top 10	0.8502	0.7496	0.7748	0.8108	0.8002	0.887 (0.885–0.890)

*Model 2 involves the features including glucose, age, male, timing of the day, interval between request and sampling, No. of outpatient visits, distance from home to hospital, division, hypertension, diabetes, coronary artery disease, stroke, statin use, and concomitant lipid testing as in Table 2.

### Impact on the prevalence of ineffective glucose measurements

On average, the prevalence of glucose measurement ≥ 126 mg/dL dropped from 14.2 to 10.1% by applying algorithm-verified FBGs over the years, and this difference was constant throughout the study period (Table 4). The prevalence of IGM dropped from 27.8% based on AC_ontological_ ≥ 126 mg/dL to 0.48% by using algorithm-verified FBGs ≥ 126 mg/dL. The difference consistently ranged between 25.9 and 28.5% from 2003 to 2018 (Table 4).

**Table 4:** Prevalence of fasting glucose ≥ 126 mg/dL and the proportion of ineffective glucose measurement (IGM) from 2003 to 2018 at China Medical University Hospital. The alphabet A to E represents the number of each condition and the proportion of each condition from B to E is derived from the ratio indicated in the brackets.

	2003–2004	2005–2006	2007–2008	2009–2010	2011–2012	2013–2014	2015–2016	2017–2018
(A) Number of patients with AC_ontological_	61,066	61,730	75,105	88,008	100,581	111,988	141,614	151,371
(B) AC_ontological_ ≥ 126 mg/dL, n (%) [B/A]	17,353 (28.4)	16,749 (27.1)	17,842 (23.8)	19,862 (22.6)	22,832 (22.7)	24,428 (21.8)	27,131 (19.2)	28,655 (18.9)
(C) IGM, n (%) [C/B]	4943 (28.5)	4771 (28.5)	5046 (28.3)	5422 (27.3)	6595 (28.9)	6676 (27.3)	7444 (27.4)	7584 (26.5)
(D) Algorithm-verified AC ≥ 126 mg/dL, n (%) [D/A]	8607 (14.1)	8458 (13.7)	9073 (12.1)	10,328 (11.7)	11,456 (11.4)	12,599 (11.3)	13,938 (9.84)	15,267 (10.1)
(E) IGM, n (%) [E/D]	4 (0.05)	56 (0.66)	38 (0.42)	58 (0.56)	56 (0.49)	71 (0.56)	74 (0.53)	89 (0.58)

## Discussion

Our findings support that fasting status can be well predicted in real-world settings by using parsimonious computation models based on ML or conventional statistical approaches in clinical practice. Using the ML model, we found that 78.0% of the 604,639 blood samples could be theoretically classified as fasting samples when we defined the fasting status as AC_ontological_ less than AC_average_. The most important features to predict fasting status were the levels of AC_ontological_, distance from the home to the hospital, age, height, and concomitant testing of serum creatinine. XGBoost yielded statistically better performance in predicting fasting status than conventional logistic regression modeling did, with an AUC of 0.892 and an F1 score of 80.5%. The prevalence of IGM decreased from 6.44 to 0.06% among those without DM history. This change is noteworthy, as the prevalence of DM was 16.6% regardless of the fasting status and 11.8% when patients with nonfasting status verified by ML algorithms were excluded from the sample. ML algorithms, such as XGBoost, may be particularly useful as their robustness to missing data, can address one of the most pervasive barriers of real-world data analysis.

In clinical practice and diabetes research, it is common to assume that AC_ontological_ is from a fasting sample in EMR^33^. Our results suggest that implementing fasting status verification algorithms based on a ML or conventional statistical approach is essential for an automated diabetes screening algorithm to better predict DM, which may help the regional and national diabetes screening policy and improve care management. There is no standardized method to assess whether patients have truly fasted before phlebotomy is performed. When patients were asked about their fasting status prior to phlebotomy in a survey study, only 50% reported having actually fasted^13^. As there is no objective biomarker to verify fasting status, the current reference standard merely relies on patients’ self-reports which are inevitably affected by recall bias. Thus, the self-report data pose persistent challenges to assessing the epidemiology of DM^13^. From the perspective of point-of-care testing, it is likely that the current literature has overestimated the prevalence and incidence of prediabetes and DM based on the EMR data, particularly the so-called “undiagnosed prediabetes or diabetes.” Information bias, specifically misclassification bias, caused by treating nonfasting glucose as fasting glucose, underestimates the effects of glucose on health outcomes. The findings of a recent study, in which six diabetes phenotyping methods in EMR were compared, suggested that solely using abnormal glucose values would overestimate the number of prevalent DM cases by approximately 1.5 times^34^. This magnitude of overestimation cannot be entirely explained by analytical variation in glucose measurement; therefore, overestimation of actual fasting status should be considered and thoroughly investigated^35^.

Our results showed that lipid profiles, except triglyceride level, were not affected by fasting status. Especially among patients without DM, levels of fasting TCHO, LDL-C, and HDL-C were counterintuitively higher than those from the nonfasting samples. This finding supports the trends of using nonfasting lipid profiles to facilitate risk assessment of atherosclerotic cardiovascular disease and assures the feasibility of our algorithm in classifying fasting status by comparing the difference between AC_ontological_ and AC_average_. Our ML approach in identifying fasting status can serve as a complementary tool to the questionnaire-based survey and enable clinicians to provide personalized instructions for fasting to patients based on their prior fasting records, thereby increasing the accuracy of the true fasting rate improving the precision in identifying DM and monitoring its control. We also observed some major contributing factors in predicting fasting status, such as distance from the home to the hospital, age, and serum creatinine level, which can provide another perspective in understanding the adherence behavior of staying in a fasting state. Furthermore, our proposed fasting status prediction algorithm helps enhance the validity of an automated diabetes phenotyping algorithm. In the entire population of CMUH-CRDR, we found that the prevalence trend of diabetes mellitus based on algorithm-verified FBGs was 11.8% lower than that based on AC_ontological_ (23.1%), and the corresponding trend of prevalent prediabetes based on algorithm-verified FBGs was also 24.1% lower than that based on AC_ontological_ (40.2%). Although the difference was not radical, the absolute misclassified number from DM to nondiabetes can be significant, depending on the population size. Indeed, due to the increasing interest and use of digital health tools to detect abnormal blood glucose levels, misclassification of nonfasting glucose measures as fasting may lead to potential overdiagnosis and treatment of patients without DM.

The concept of IGM is worthy of broader discussion as it stands for a measurement of FBG that did not change the clinical course of glucose metabolism even when the level was greater than 126 mg/dL among patients without a history of DM. Several reasons could help explain this observation, such as clinician knowledge of the nonfasting status or a missed interpretation of the result. Nonetheless, a potential consequence of IGM is missing the detection of diabetes, leading to complications and increased healthcare utilization in the long run. Failing to obtain a truly FBG may be problematic for diabetes screening. Our proposed algorithms drastically reduced the proportion of IGM, supporting their use in the real-world care flow to trigger actionable screening of diabetes. These algorithms also help generate a warning upon detecting the discrepancy between AC_ontological_ and algorithm-verified nonfasting glucose, which could serve as a checkpoint and reminder in the automatically digital phenotyping process for DM screening. Future research on clinical effectiveness and automatic fasting status prediction implementation in the flow of digital diabetic phenotyping systems is necessary to strengthen the public health impact.

The present study has several limitations. First, the actual fasting status of the patients was not available. However, it is challenging, if not impossible, to obtain the actual fasting status. We assumed that AC_ontological_ should be less than AC_average_ in the fasting status among outpatients with stable dietary habits and a steady level of carbohydrate metabolism. In the crude analysis, we found that patients from the Health Management Center were more likely to be in the fasting state before phlebotomy. This observance corresponds to our clinical experience, where patients who were relatively healthy and willing to attend health checkups typically have a higher motivation to provide fasting samples. Specifically, patients who undergo health checkups usually receive detailed instructions for fasting^36^. Furthermore, over 93% of FBGs obtained from patients prepared for a pan-endoscopy were accurately classified as AC_theoretical_. Second, the algorithm was developed in a tertiary hospital under universal health care coverage; thus, it may not be generalizable to other settings. Further research with additional data from different populations is required to train and solidify our proposed algorithm. More importantly, integrating our algorithm into the clinical workflow is critical to verify its performance in the real-world setting.

## Conclusions

To the best of our knowledge, this is the first attempt at using a ML approach to evaluate the reliability of fasting samples in a large tertiary hospital. Only 65.3% of ontologically AC samples could be classified as algorithm-verified fasting status. Despite its moderate performance in predicting the fasting status among outpatients, our algorithms provide an innovative approach to clean medical data and facilitate true fasting BG detection. Notably, this study has introduced an essential step towards establishing automated phenotyping in EMR for effective diabetic screening and more accurate estimation of the global and local epidemiology of DM.

## Supplementary Information

Supplementary Tables.

## Data Availability

Restrictions apply to the availability of some or all data generated or analyzed during this study to preserve patient confidentiality or because they were used under license. The corresponding author will on request detail the restrictions and any conditions under which access to some data may be provided.
