# Supplementary material for: Application of machine learning methods for the prediction of true fasting status in patients performing blood tests
Source: Sci Rep. 2022 Jul 13;12:11929. doi: 10.1038/s41598-022-15161-2 (PMC9279373; doi:10.1038/s41598-022-15161-2)
Supplement: Supplementary file 1 — Supplementary Tables. [file 41598_2022_15161_MOESM1_ESM.docx]

**Supplementary Material**

**Supplementary Table 1.** Range of the parameters in grid search for XGBoost and CatBoost.

**Supplementary Table 2.** Features selected in XGBoost classifier for classification of theoretical fasting and nonfasting status (total features, n = 67).

**Supplementary Table 3.** Proportion of missingness for features selected in the balanced dataset.

**Supplementary Table 4**. Comparison of performance of determining fasting status by XGBoost, CatBoost, H2O Ensemble and multiple logistic regression models in the training dataset.

**Supplementary Table 1.** Range of the parameters in grid search for XGBoost and CatBoost.

|  | **Max depth** | **Learning rate** | **Number of estimators** | **Bagging temperature** | **Gamma** | **Subsample** | **Colsample by tree** |
| --- | --- | --- | --- | --- | --- | --- | --- |
| **XGBoost** | 3-8 | 0.01-0.1 | 50-300 |  | 0.5-2.0 | 0.6-1.0 | 0.6-1.0 |
| **CatBoost** | 5-10 | 0.001-0.1 | 20-300 | 3-10 |  |  |  |

**Supplementary Table 2.** Features selected in XGBoost classifier for classification of theoretical fasting and nonfasting status (total features, n = 67).

| **Categories of features** | **Description** |
| --- | --- |
| **Demographics** | Age, sex, distance from home to hospital, height, weight, body mass index |
| **Comorbidity** | Hypertension, diabetes, coronary artery disease, heart failure, peripheral vascular disease, stroke |
| **Medication** | **Cardiovascular system:** Nitroglycerin, nitrocontin, isosorbide dinitrate, isosorbide-5-mononitrate, angiotensin- converting enzyme inhibitors, angiotensin receptor blockers, calcium channel blocker, hydralazine, diuretics, alpha / beta blocker, alpha-1 blocker, alpha-2 agonist, beta blocker, aspirin, dipyridamole, clopidogrel, ticlopidine, ticagrelor, cilostazol, wafarin, non-vitamin k antagonist oral-anticoagulant, heparin  **Anti-diabetic agents:** Insulin, metformin, sulfonylurea, thiazolinedione, alpha glucosidase inhibitors, dipeptidyl peptidase-4 inhibitor, glinide, sodium-glucose co-transporter 2 inhibitor  **Lipid-lowering agents:** Fibrates, statins |
| **Healthcare utilization** | The clinical division where the glucose test was requested, No. of outpatient visits |
| **Blood test** | Glucose, hemoglobin, red blood cell count, total cholesterol, HDL, LDL, triglyceride, BUN, SCr, serum sodium, serum potassium, albumin, AST, ALT, uric acid |
| **Blood test time** | Sampling timing of the day, interval between request and sampling |
| **Urinalysis** | pH, specific gravity |
| **Data augmentation** | Estimated blood osmolality, BUN/SCr, AST/ALT, Hemoglobin /red blood cell count, Serum sodium / serum potassium, |

***Abbreviations***: LDL, low-density lipoprotein; HDL, high-density lipoprotein; BUN, blood urea nitrogen; SCr, serum creatinine; AST, aspartate aminotransferase; ALT, alanine transaminase

**Supplementary Table 3.** Proportion of missingness for features selected in the balanced dataset.

|  | **non-Diabetes**  **(n = 118383)** | | **Diabetes**  **(n = 241019)** | |
| --- | --- | --- | --- | --- |
| **Varibles** | Available n | Missing rate, % | Available n | Missing rate, % |
| Body mass index | 50389 | 57.4 | 80243 | 66.7 |
| Distance to hospital, km | 110655 | 6.5 | 237811 | 1.33 |
| Biochemical variables |  |  |  |  |
| Red blood cell count, x10^6^/μL | 50187 | 57.6 | 14398 | 94.0 |
| Hemoglobin, g/dL | 60078 | 49.3 | 25539 | 89.4 |
| Total Cholesterol, mg/dL | 89664 | 24.3 | 115610 | 52.0 |
| LDL, mg/dL | 85369 | 27.9 | 126903 | 47.4 |
| HDL, mg/dL | 75309 | 36.4 | 88027 | 63.4 |
| Triglyceride, mg/dL | 91872 | 22.4 | 119958 | 50.2 |
| BUN, mg/dL | 60080 | 49.2 | 34391 | 85.7 |
| SCr, mg/dL | 94258 | 20.4 | 137714 | 42.9 |
| Serum sodium, mmol/L | 28644 | 75.8 | 26212 | 89.1 |
| Serum potassium, mmol/L | 35452 | 70.1 | 42004 | 82.6 |
| AST, IU/L | 63715 | 46.2 | 30207 | 87.5 |
| ALT, IU/L | 89565 | 24.3 | 103881 | 56.9 |
| Uric acid, mg/dL | 72427 | 38.8 | 58639 | 75.7 |
| Albumin, g/dL | 49063 | 58.6 | 23569 | 90.2 |
| Estimated blood osmolality | 24363 | 79.4 | 19566 | 91.9 |
| Urine specific gravity | 41168 | 65.2 | 17425 | 92.8 |
| Urine pH | 41168 | 65.2 | 17425 | 92.8 |

***Abbreviations***: LDL, low-density lipoprotein; HDL, high-density lipoprotein; BUN, blood urea nitrogen; SCr, serum creatinine; AST, aspartate aminotransferase; ALT, alanine transaminase

**Supplementary Table 4**. Comparison of performance of determining fasting status by XGBoost, CatBoost, H2O Ensemble and multiple logistic regression models in the training dataset.

| **Algorithm/**  **Modeling strategy** | **Feature** | **Sensitivity** | **Specificity** | **Precision** | **F1-score** | **Accuracy** | **AUC** |
| --- | --- | --- | --- | --- | --- | --- | --- |
| **Parsimonious modeling** |  |  |  |  |  |  |  |
| Logistic regression | Model 2^*^ | 0.7616 | 0.807 | 0.7993 | 0.7799 | 0.7840 | 0.867 (0.866-0.869) |
| XGBoost | Model 2^*^ | 0.8012 | 0.8705 | 0.814 | 0.8413 | 0.8358 | 0.921(0.920-0.922) |
| CatBoost | Model 2^*^ | 0.7740 | 0.8379 | 0.7876 | 0.8120 | 0.8060 | 0.894 (0.893-0.895) |
| H2O Ensemble | Model 2^*^ | 0.7861 | 0.9517 | 0.8164 | 0.8789 | 0.8689 | 0.958 (0.958-0.959) |
| **Full modeling** |  |  |  |  |  |  |  |
| XGBoost | 67 | 0.8224 | 0.8837 | 0.8326 | 0.8574 | 0.8531 | 0.934 (0.933-0.935) |
| CatBoost | 67 | 0.7639 | 0.8668 | 0.7858 | 0.8243 | 0.8153 | 0.903 (0.902-0.904) |
| H2O Ensemble | 67 | 0.7969 | 0.9277 | 0.8203 | 0.8707 | 0.8623 | 0.946 (0.947-0.946) |
| **Feature selection modeling** |  |  |  |  |  |  |  |
| XGBoost | Top 45 | 0.8156 | 0.8782 | 0.8264 | 0.8515 | 0.8469 | 0.930 (0.929-0.931) |
| XGBoost | Top 35 | 0.7919 | 0.8598 | 0.8051 | 0.8315 | 0.8258 | 0.912 (0.911-0.912) |
| XGBoost | Top 25 | 0.7710 | 0.8407 | 0.7881 | 0.8135 | 0.8061 | 0.893 (0.891-0.896) |
| XGBoost | Top 10 | 0.7522 | 0.8549 | 0.7752 | 0.8131 | 0.8036 | 0.891 (0.890-0.892) |

* Model 2 involves the features including glucose, age, male, timing of the day, interval between request and sampling, No. of outpatient visits, distance from home to hospital, division, hypertension, diabetes, coronary artery disease, stroke, statin use, and concomitant lipid testing as in Table 2.
